# Polydots, soft nanoparticles, at membrane interfaces†

**DOI:** 10.1039/d3ra02085a

**Published:** 2023-06-26

**Authors:** Sidath Wijesinghe, Christoph Junghans, Dvora Perahia, Gary S. Grest

**Affiliations:** a Department of Chemistry, Clemson University Clemson South Carolina 29634 USA dperahi@g.clemson.edu; b Los Alamos National Laboratory Los Alamos New Mexico 87545 USA; c Sandia National Laboratories Albuquerque New Mexico 87185 USA gsgrest@sandia.gov

## Abstract

Soft nanoparticles (NPs) are emerging candidates for nano medicine, particularly for intercellular imaging and targeted drug delivery. Their soft nature, manifested in their dynamics, allows translocation into organisms without damaging their membranes. A crucial step towards incorporating soft dynamic NPs in nano medicine, is to resolve their interrelation with membranes. Here using atomistic molecular dynamics (MD) simulations we probe the interaction of soft NPs formed by conjugated polymers with a model membrane. These NPs, often termed polydots, are confined to their nano dimensions without any chemical tethers, forming dynamic long lived nano structures. Specifically, polydots formed by dialkyl *para* poly phenylene ethylene (PPE), with a varying number of carboxylate groups tethered to the alkyl chains to tune the interfacial charge of the surface of the NP are investigated at the interface with a model membrane that consists of di-palmitoyl phosphatidylcholine (DPPC). We find that even though polydots are controlled only by physical forces, they retain their NP configuration as they transcend the membrane. Regardless of their size, neutral polydots spontaneously penetrate the membrane whereas carboxylated polydots must be driven in, with a force that depends on the charge at their interface, all without significant disruption to the membrane. These fundamental results provide a means to control the position of the nanoparticles with respect to the membrane interfaces, which is key to their therapeutic use.

## Abstract 2

Soft nanoparticles (NPs) are emerging candidates for nano medicine, particularly for intercellular imaging and targeted drug delivery.

## Graphical abstract

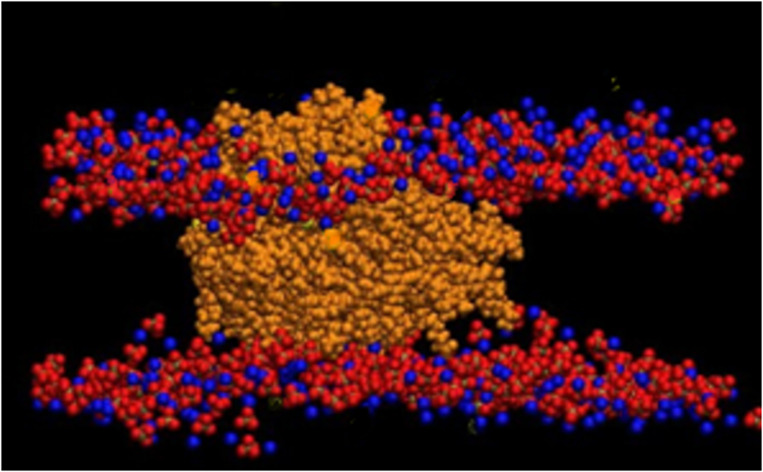

## I. Introduction

Nanoparticles (NPs) exhibit innovative pathways for intracellular imaging trackers, and targeted drug-delivery systems.^1–8^ Their diverse and versatile chemistries include inorganic particles, bare or grafted with organic functionalities, and soft-organic based particles. The therapeutic promise of NPs depends on their ability to reach the targeted location with minimal disruption to the bio-system.^1–7^ Soft NPs that are often similar in stiffness to that of membranes, internally dynamic, and responsive, constitute such particles. They typically consist of assemblies of molecules such as micelles and vesicles of both surfactants and polymers and are highly responsive to their environment. However, these self-assembled systems often lack the mechanical stability required for translocation across membrane barriers. The immense potential of nano medicine, coupled with a proof of concept of the use of soft NPs in nano medicine, has propelled the search for soft, responsive NPs, able to transcend through membranes.

A new class of NPs is formed by conjugated polymers confined into nano-dimensions without any chemical crosslinks, which form long-lived far-from-equilibrium particles.^9–12^ These are often termed polydots or conjugated-polymer-nano particles (CNPs). They are inherently highly luminescent,^13–16^ and emit at frequencies that are not absorbed by membranate, and hence they could be *in vivo* trackable. The glassy nature of the polymer under confinement, drives their stability, where the absence of chemical tethers permits potential responsiveness. Their far-from-equilibrium nature makes them potentially tunable responsive NPs, whose light-emitting characteristics change with the backbone conformation.^17,18^ Their chemical structure enables encoding the NPs' interface with specific recognition groups. Further, the luminescent polymer can be co-confined with cargo such as therapeutics into NPs and remain stable.^19^ Conjugated polymers are often substituted by alkyl side chains that enable their solubility. When confined to their NP configuration, these side chains whose stiffness is similar to that of membranes, reside at the particle interface. The polymer backbone remains dynamic on the microsecond time scale,^20,21^ in contrast to coated inorganic nanoparticles.

Here using all-atom molecular dynamics simulations (MD), we probe the interrelation between a polydot^20–23^ and a DPPC lipid membrane, all immersed in water, to attain molecular insight into the penetration of polydots into membranes, as the interfacial charge of the polydots is varied. We find that the polydots are able to translocate across membranes, retaining their shapes and their interfacial charge determines their location with respect to the membrane interface, facilitating control of the location of the polydots for targeting specific nanomedicine uses.^16^

There have been ample of examples for the use of soft nanoparticles, such as polydots, for different nano medicine applications from enhance efficiency of photo therapy to embedding sugar-monitoring sensors. However, universal principles that will allow tailoring polydots for targeted nano medicine applications have not been established. The challenge lies in the large number of characteristics of NPs,^24^ that affect their interactions with membranes and their translocation into cells, including size,^25–29^ shape,^30–32^ hydrophobicity,^33^ charge,^34–40^ and surface chemistry.^25,41–50^

Most numerical studies on NPs at the interface of membranes have focused on either bare NPs^28,51,52^ or gold NPs grafted with short ligands.^29,35,36,38,43,44,46,47,50,53–55^ Simulations have shown that electrostatic interactions between surface charged gold NPs and DPPC molecules induce local transitions in fluid bilayers resulting in adhesion of charged NPs to the membrane. Besides NP hydrophobicity, their size relative to the membrane is found to be an important factor in determining the NP–membrane interactions which control the nonspecific NP uptake into cells and translocation across the membrane.^25–29,31^ These particles, however, often disrupt the membranes. In contrast, soft NPs^56^ that can morph through the translocation, without disrupting the membrane would pose significant advantage for use in nano-medicine.

**Fig. 1:**
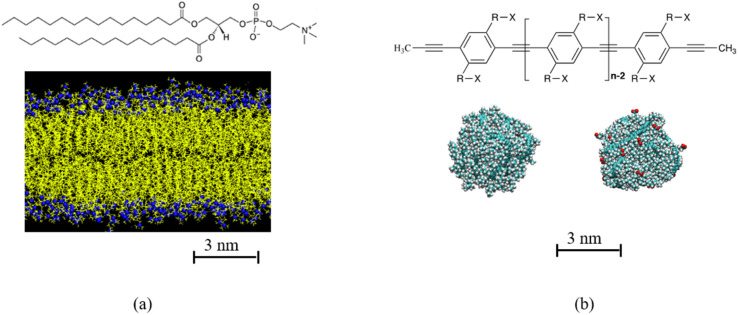
(a) Chemical structure of di-palmitoyl phosphatidylcholine (DPPC), DPPC membrane. Hydrocarbon tail of DPPC is shown in yellow, N and P atoms in DPPC head group is shown in blue. (b) Chemical structure of poly *para* phenyl ethylene (PPE), where in this study R is C_8_H_16_ and X can be either CH_3_ or COO^−^Na^+^. Example of polydots with *n* = 60 and *f* = 0 and 0.4 are shown in (b). The carboxylate groups for *f* = 0.4 polydot is shown in red.

The current study investigates the effects of two parameters, the hydrophobicity of the interface of the polydots and their size on their translocation through a DPPC membrane. The polydot that consists of dinonyl poly *para* phenylene ethynylene (PPE)^57^ with a fraction of the nonyl chains are end terminated by carboxylate groups. As the polydots reside in water, the carboxylate groups migrate to the interface, and the degree of hydrophobicity at the polydot interface is determined by the number of the carboxylate decorated side chains.^22^ The chemical structure of DPPC and PPEs are shown in Fig. 1. In equilibrium PPE chains are extended objects with a large persistence length independent of solvent quality.^58^ Confined to nano dimensions, they remain collapsed experimentally for extended times.^9–12^ We show that while the polydots locally fluctuate, they remain stable as they enter the membrane. We further provide correlations between the interfacial charge and particle size and their penetration pathways, information on atomistic level, which is attainable only through the high resolution provided by probing the NP–membrane complexes.

## II. Model and methodology

Polydots are formed experientially by trapping the polymers in droplets of good solvent that is dripped into water, which is a poor solvent for the PPE chains.^9–12^ As the good solvent evaporates under sonication, the polymer remains in a long-lived trapped state. To model the experimental process, an isolated PPE chain is dissolved in a good solvent tetrahydrofuran (THF) and encapsulated in a spherical cavity where only the atoms of the PPE chain interact with the cavity wall by a purely repulsive harmonic potential.^20–22^ Good solvent evaporation is mimicked by decreasing the size of the confining cavity until the density of the polydot is comparable to that of a PPE melt. The spherical cavity is removed and the polydot is placed in water and allowed to relax for up to 60 ns before introducing them to the lipid bilayer.

A dinonyl PPEs with *n* the number of monomers *n* = 60 and 120 monomers containing ∼3960 and 7930 atoms, formed polydots with diameters of ∼3.2 nm and 4.6 nm respectively. A fraction of the nonyl groups, –(CH_2_)_8_CH_3_, substituted by nonylate –(CH_2_)_8_COO^−^ side chains. The polydot functionalization is controlled by randomly varying the fraction *f* from of aromatic rings which have one side chain which is carboxylate terminated from *f* = 0, 0.1, 0.2 and 0.4 for *n* = 60. For *n* = 120, *f* = 0 and 0.4. Charge neutrality is maintained by introducing one Na^+^ counterion per COO^−^ group.^23,59^ In water, the polydots remain collapsed with a uniform dense core of density of ∼1.0 g cm^−3^ with an interfacial width of approximately 1 nm independent of *f*. Water hardly penetrating into the polydot. The interface of the polydot is dominated by the side chains with the majority of the carboxylates reside at the interface for *f* > 0.^22,23^ About 90% of the carboxylates are at the surface of the polydot. As practically all the Na^+^ ions are hydrated (*i.e.* non-condensed), the polydots for all *f* > 0 are charged.

The impact of the carboxylate groups was quantified through calculating the three eigenvalues (*λ*_1_ < *λ*_2_ < *λ*_3_) of the radius of gyration tensor and average root mean square radius of gyration 〈*R*_g_^2^〉^1/2^. For the larger polydot (*n* = 120) in water at 323 K, 〈*R*_g_^2^〉^1/2^ increases slightly from 2.3 nm for *f* = 0 to 2.5 nm for *f* = 0.4. The rations of *λ*_3_/*λ*_1_ and *λ*_2_/*λ*_1_ provide a measure of divergence from sphericity where for a fully spherical object these ratios are equal to 1. For *f* = 0, *λ*_3_/*λ*_1_ = 1.4 and *λ*_2_/*λ*_1_ = 1.3. With increasing *f* to 0.4, *λ*_3_/*λ*_1_ = 1.7 and *λ*_2_/*λ*_1_ = 1.5. No measurable changes occur for the overall size or degree of a-sphericity are observed when the polydot is in contact with the membrane.

The PPEs chains and solvents were modeled fully atomistically using the optimized potentials for liquid simulations-all atoms (OPLS-AA).^60,61^ Simulations of polydots prior to their introduction into the bilayers were performed using the large-scale atomic/molecular massively parallel simulator (LAMMPS) molecular dynamics simulations code.^62^ Further details of the simulations of single polydots can be found in ref. 22.

The initial coordinates for DPPC membrane are downloaded from https://lipidbook.bioch.ox.ac.uk/. That system includes 128 DPPC molecules and 3840 water molecules in a cell with dimensions of 6.30 × 6.41 × 6.68 nm^3^. The mid plane of the membrane is defined as the *XY* plane and *Z* axis is perpendicular to the bilayer surface. *Z* = 0 corresponds to the center of the DPPC membrane, which has a thickness of 4.48 nm. Initial configuration is replicated once in *x* and *y* directions, to obtain a membrane large enough to accommodate the polydot. The resulting membrane contains 512 DPPC molecules. To allow space for the membrane to expand, in the presence of the polydot, a lipid ribbon which mimics experimental membranes, is used. The ribbon is periodic in *y* direction and open in the *x* direction^26,43^ and is obtained by removing the periodic boundary conditions in the *x* direction and extending the simulation in the *X* direction by 8 nm. The resulting ribbon is equilibrated in water for 100 ns before introducing the polydot. The equilibrated surface area per DPPC molecule is 0.63 nm^2^ per DPPC, the same as the original membrane. Water molecules were modeled using TIP3P water model.^63^ During the equilibration some lipid molecules migrated to the edge of the ribbon forming curved edges to minimize the exposure of the hydrophobic tails of the DPPC molecules to water.

Simulations of the lipid membrane and polydot–membrane complex were performed using GROMACS 4.6.5.^64,65^ Temperature is maintained at 323 K, which is above the gel-to-liquid phase transition temperature of DPPC using the Nose–Hoover thermostat.^66,67^ System pressure was maintained at 1 bar using the Berendsen semi isotropic pressure coupling scheme^68^ with an isothermal compressibility of 4.5 × 10 ^−5^ bar ^−1^. Simulation time step was set to 2 fs. Electrostatic interactions were calculated using the particle-mesh Ewald (PME) with a Fast Fourier Transform (FFT) grid spacing of 0.12 nm. The Lennard-Jones and short-range electrostatic cutoff was set to 1.0 nm. LINCS^69^ algorithm is used to constrain the bonds.

The polydot–membrane system was simulated in two ways, first by mimicking spontaneous interactions and then by pulling the polydots into the center of the membrane. To mimic a spontaneous ingestion, an equilibrated polydot was placed at the surface of the lipid membrane by forming a void in water near the water–membrane interface. The resulting configuration contains ∼44 800 atoms in a 20.64 × 12.67 × 15.84 nm^3^ box. These dimension in the *Z* direction is large enough that the polydot does not interact with the other surface through the periodic boundary conditions. The area of lipid membrane is significantly larger than the diameter of the polydots. After the initial energy minimization and equilibration, we restrained the center of mass (COM) of the polydot near the membrane/water interface and equilibrated for 50 ns followed by their release.

## III. Results and discussion

**Fig. 2:**
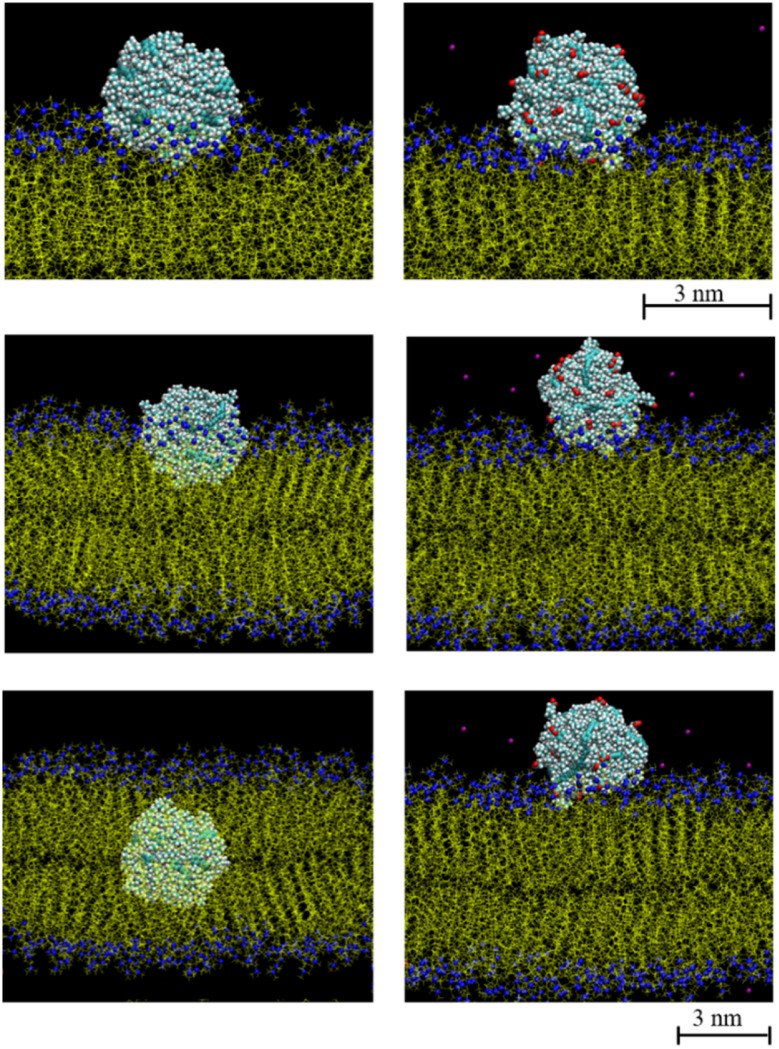
Time evolution of *n* = 60, *f* = 0 (left column) and 0.4 (right column) polydots interacting with the lipid membrane at times *t* = 0 (top), *t* = 75 ns (middle), and *t* = 200 ns (bottom). N and P atoms in DPPC head group is shown in blue. Hydrocarbon tails of DPPC molecules are shown in yellow. Carboxylate groups in polydots with *f* = 0.4 are shown in red and Na^+^ are shown in purple.

Neutral polydots and those decorated with carboxylates were placed at the DPPC membrane interface. Following a short equilibration of the polydot at the membrane interface, the polydot was released and the system followed with time as shown in Fig. 2. The top two images are visualized at 10 ns following the release of the polydot. The neutral (non-charged) *f* = 0 polydot penetrates the membrane whereas the *f* = 0.4 polydot remains adsorbed at the membrane surface. These results are consistent with prior studies that have shown that charged NPs tend to absorb to the membrane surface,^44,57,58^ though the mechanism remains an open question. As seen in the images on the left for *f* = 0, the polydot has only a minimal effect on the DPPC membrane. After a distance only 1 nm or less the DPPC molecules are unperturbed as measured by their orientational order relative to the plane of the membrane. The area per head group away from the polydot is unchanged. Movies of interpenetration of a polydot with *n* = 120 for *f* = 0 into the membrane is included in the ESI,† as well as a movie for the *n* = 120/*f* = 0.4 polydot which remains at the water/membrane interface. The movies show that while the polydot remains collapsed, it fluctuates in size and slightly rotates.

**Fig. 3:**
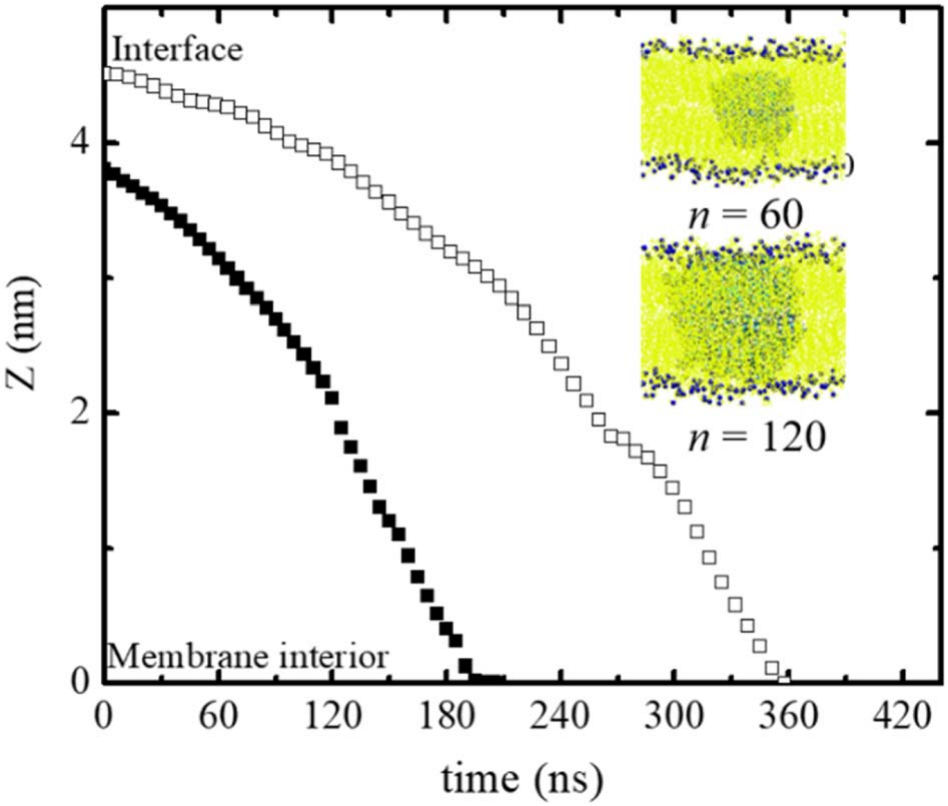
Position of the center of mass of polydot with *n* = 60 (closed symbols) and *n* = 120 (open symbols) as a function of time. *Z* = 0 corresponds to the center of mass of the membrane. Time *t* = 0 is when the polydots are released.

The size of the nanoparticles also plays an important role in the penetration of the polydots into the membrane. Here the penetration of polydots with *f* = 0 polydots, one with *n* = 60 and diameter ∼ 3.2 nm, whose dimensions are smaller than the membrane thickness and a larger one, with *n* = 120 and diameter ∼ 4.6 nm, whose dimension is comparable to the membrane thickness, are followed as a function of time as shown in Fig. 3. At the initial stage, the smaller polydot penetrates the membrane faster than the larger one. Within the hydrophobic, inner layer, the rate of penetration is comparable for the two polydots. As the interface of the neutral polydots consist predominantly of alkane chains, which are similar in chemical structure to the DPPC hydrophobic tail, there is essentially no energy barrier for the neutral polydot to penetrate the membrane.

**Fig. 4:**
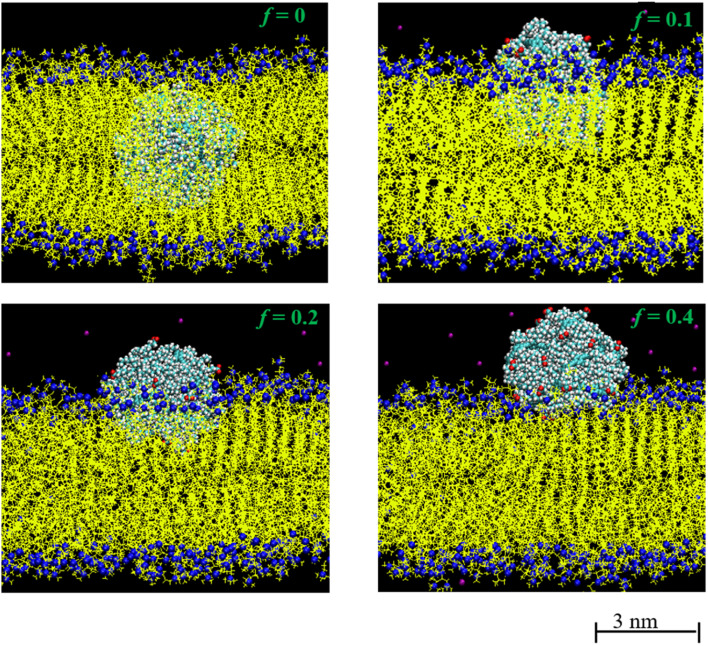
Equilibrium positions of the polydot with *f* = 0, *f* = 0.1, *f* = 0.2 and *f* = 0.4. N and P atoms in DPPC head group are shown blue. Hydrocarbon tails of DPPC molecules is shown in yellow. Carboxylate groups in polydots are shown in red and Na^+^ are shown in purple.

Further insight into the membrane–polydot interrelation was obtained by pulling the polydots into the membrane by applying a weak, constant force of a 500 kJ mol^−1^ nm^−1^ to their COM. This force is chosen to minimize the deformations of the membrane. With this force, it takes about 10 ns to pull the polydot to the center of membrane. After the polydot reached the center of the membrane, the force was released and the position of the polydots followed until no changes were observed. Fig. 4 shows the final positions of *n* = 60 polydots for different *f* values.

**Fig. 5:**
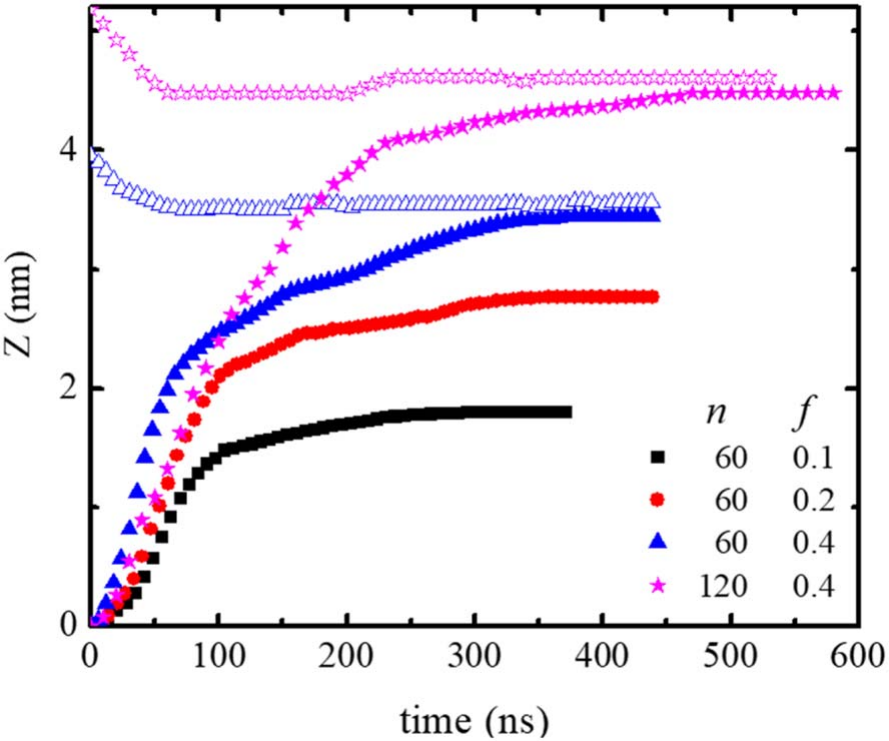
Distance between the COM of the DPPC lipid membrane and polydot COM as a function of time after polydot is released from the center of the DPPC membrane (solid) and from membrane interface (open). The water/DPPD interface is at *Z* ∼ 2.24 nm.

The neutral, *f* = 0, polydot remains in the hydrophobic region of the membrane, while polydots with *f* = 0.4 migrate to the hydrophilic, outer interface. The distance of the COM of the polydot from the center of the membrane *Z* as a function of time after the force removed is shown in Fig. 5. Remarkably, polydots with *f* = 0.1 and *f* = 0.2 assume intermediary positions across the membrane hydrophilic layers, with the center of mass of the polydot with *f* = 0.1 slightly below and with *f* = 0.2 slightly above the water/DPPC interface (*Z* ∼ 2.24 nm). The final positions of the polydots do not depend on their starting position, inside or at the interface of the membrane.

**Fig. 6:**
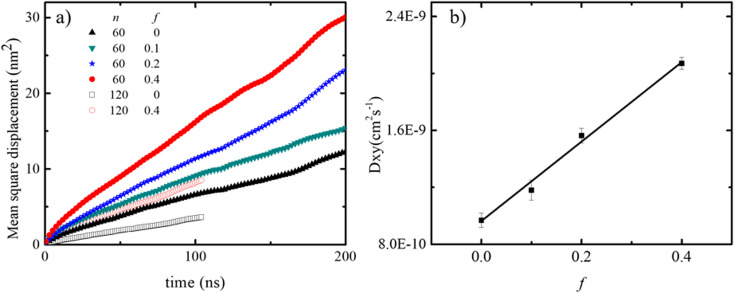
(a) MSD for *n* = 60 and 120 polydots and (b) lateral diffusion coefficient *D*_*XY*_ of *n* = 60 polydots as a function *f*.

The lateral motion of the polydots in their equilibrium position was followed by calculating their mean square displacement within the plane of the membrane plane as shown in Fig. 6a. Lateral movement of polydots are restricted by the neighboring DPPC molecules as observed in the movie presented in ESI Fig. 1 for a neutral polydot of size *n* = 120. The more they are submerged into the membrane, the greater the hindrance to their motion becomes. This results in a lateral diffusion constant *D*_*XY*_ which increases linearly with *f* as shown in Fig. 6b. For comparison, *D*_*xy*_ = 1.1 × 10^−7^ cm^2^ s^−1^ for DPPC molecules away from the polydot.

**Fig. 7:**
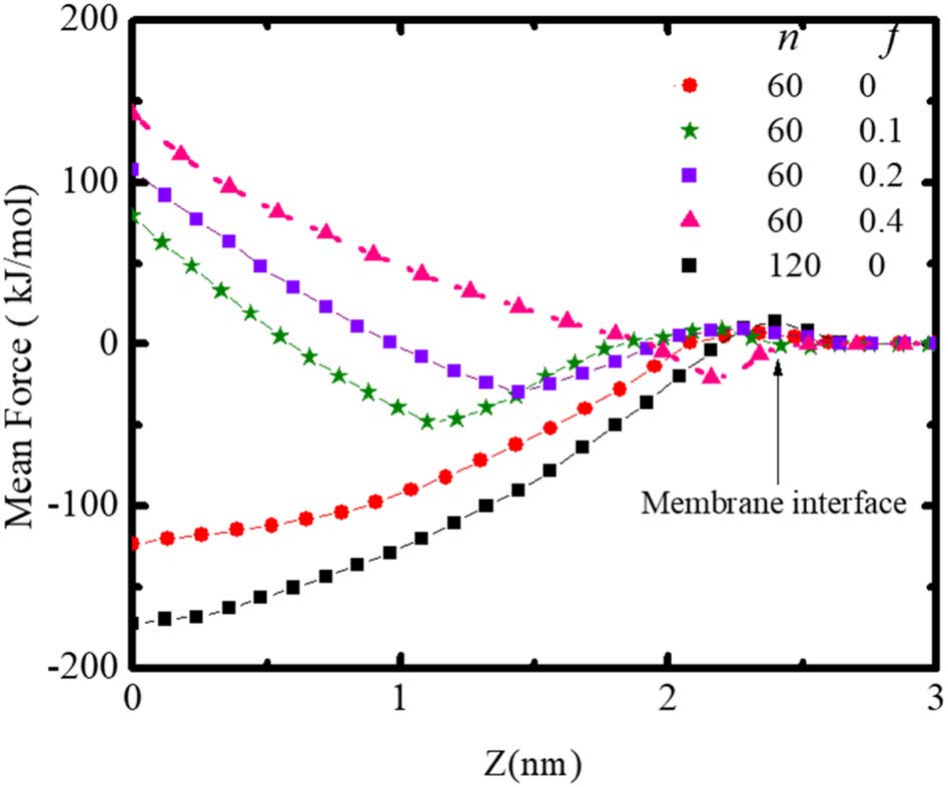
Mean force on the polydot as a function of distance from the COM of the lipid ribbon for polydots with indicated *f* and *n*.

The force required to maintain the polydot at different distances *Z* from the center of the membrane depends on both the polydot size and charge as shown in Fig. 7. The force to maintain the polydot a given *Z* is obtained by holding the COM of the polydot at a fixed value of *Z* and averaging the force acting on the polydot. For each simulation, the force was averaged over 5 ns after an equilibrium of 3 ns. The mean force is defined as negative if it pulls the polydot toward the DPPC membrane center. For *f* = 0 the minimum force is at the center of the membrane center, which we attribute to the affinity between the lipid tails and hydrophobic polydot surface.

This provides the driving force for *f* = 0 polydots to spontaneous ingestion. For polydots with *f* = 0.1 and 0.2 there is an initial negative force with a minimum at the distance comparable with their equilibrium locations within the membrane, followed by a gradually increasing force towards the hydrophobic membrane center, resulting in interstitial final position of these polydots. Finally, for *f* = 0.4 the minimum force is at the interface with significant repulsion in the hydrophobic region, hindering the penetration of the polydots.

## IV. Conclusions

The interrelation between PPEs polydots and a DPPC membranes were studied as a function of their size and charge. The polydots were smaller or comparable to the membrane dimensions and the charge was controlled by the degree of carboxylation termination of the alkyl side chains. With the carboxylates residing predominantly at the polydot interface, and the Na^+^ counterions being hydrated, the polydots become negatively charged. Independent of charge, we find that the polydots remain in their collapsed state as they interact with the membrane. Neutral polydots with carboxylate fraction *f* = 0 spontaneously penetrate the membrane. The carboxylated polydots reside across the hydrophilic layer, partially immersed in water and partially in the membrane where the location normal to the membrane depends on the degree of *f*. We find that even small fraction of carboxylates is sufficient to prevent penetration of the polydots into the membrane. As the total forces operating on the polydots include hydration forces, as well as hydrophobic and hydrophilic ones, the equilibrium position is a result of a balance of all interactions in the system.

## Conflicts of interest

There are no conflicts of interest to declare.

## Supplementary Material

RA-013-D3RA02085A-s001

RA-013-D3RA02085A-s002
